# Toward a high-throughput in vitro model for estimating vitreous humor permeability of topically applied drugs

**DOI:** 10.1038/s41598-025-93425-3

**Published:** 2025-03-13

**Authors:** Anna Vincze, Eszter Simon, Gábor Koplányi, József Gergely Stankovits, Diána Balogh-Weiser, Benjámin Gyarmati, Zoltán Zsolt Nagy, György T. Balogh

**Affiliations:** 1 Department of Pharmaceutical Chemistry, Semmelweis University, Hőgyes Endre Street 9, Budapest, H-1092 Hungary; 2 Center for Pharmacology and Drug Research & Development, Semmelweis University, Üllői Street 26, Budapest, H-1092 Hungary; 3 Department of Electronics Technology, Faculty of Electrical Engineering and Informatics, Budapest University of Technology and Economics, Műegyetem Quay 3, Budapest, H-1111 Hungary; 4 Department of Organic Chemistry and Technology, Faculty of Chemical Technology and Biotechnology, Budapest University of Technology and Economics, Műegyetem Quay 3, Budapest, H-1111 Hungary; 5 Department of Physical Chemistry and Materials Science, Faculty of Chemical Technology and Biotechnology, Budapest University of Technology and Economics, Műegyetem Quay 3, Budapest, H-1111 Hungary; 6 Department of Ophthalmology, Semmelweis University, Mária Street 39, Budapest, H-1085 Hungary

**Keywords:** Posterior segment of the eye, Ocular permeability, In vitro permeability model, PAMPA, Vitreous humor, Vitreous substitutes, Sodium hyaluronate, Medical research, Drug development, Preclinical research, Drug discovery, Drug delivery, Drug screening

## Abstract

The vitreous humor of the eye presents a crucial target for posterior segment therapy due to its proximity to the retina and relatively easy accessibility. Although intravitreous injections have long been the primary method for treating posterior segment disorders, recent successes in non-invasive topical treatments have led to the exploration of alternative administration routes. The objective of our study is to establish a platform for a Parallel Artificial Membrane Permeability Assay (PAMPA) model that mimics the posterior segment, modelling the permeation process of compounds applied topically on the eye. The study demonstrates the acceptor-phase effects of sodium hyaluronate and agar on passive permeability, while applying a previously published vitreous humor-mimetic material. Physicochemical similarities of the acceptor phase media and freshly excised porcine vitreous humor further support the applicability of the mimetic material, based on viscosity and zeta potential measurement. As a result, a new concept is introduced for measuring posterior segment permeability, with potential for future high throughput screening applications.

**Supplementary Information:**

The online version contains supplementary material available at 10.1038/s41598-025-93425-3.

## Introduction

Ophthalmic diseases related to the posterior segment of the eye (age-related macular degeneration, proliferative vitreoretinopathy, diabetic macular oedema, endophthalmitis) are the leading causes of vision loss worldwide, however the therapeutic treatment of the posterior still poses a great challenge^1,2^. Target tissues include the vitreous body, the retina, and the choroid – and while there are more strategies to reach these tissues, none of them comes without drawbacks. The vitreous body is a ~ 4 g weighting, gel-like tissue filling the cavity between the lens and the retina, mainly consisting of water (99.9%), ions, hyaluronic acid and collagen^3^. Invasive therapies (mainly intravitreal injections) show the highest success rate with the lowest systemic absorption, although they often cause retinal detachment, elevated intraocular pressure, glaucoma, choroidal or vitreous haemorrhage or lead to cataract formation^4^. Another problem is the lack of patient compliance, as these therapies are not only invasive and thus uncomfortable, but they also require repeated administration by trained personnel^2^. Some intravitreal inserts offer prolonged drug delivery, but their placement into the eye is still invasive (via injection or surgery) and the non-biodegradable ones require removal at some point^4^. A further alternative is systemic administration, as drugs can enter the eye via the choroid from the blood circulation, however the blood-retina-barrier, and the efflux pumps of the retinal pigmented epithelium greatly limit the amount of drug reaching the posterior segment, thus higher doses are required with the increased risk of systemic adverse effects^5^.

The most convenient route would be topical administration offering better patient compliance and fewer side effects. In contrast with the onetime beliefs, nowadays it seems that eye drops can actually achieve therapeutic drug levels in the posterior segment (it was proved for aqueous eye drops containing dorzolamide^6^, brimonidine^7^, betaxolol^8^, netarsudil^9^, memantine^10^). Also, more and more unconventional eye drops have been demonstrated to be beneficial in the treatment of posterior segment disorders (e.g. liposomes, solid lipid nanocarriers, lipid DNA-nanoparticles, cyclodextrin-nanoparticles, chitosan oligosaccharide nanomicelles and chitosan-coated liposomes to deliver bevacizumab^11^, indomethacin^12^, brimonidine^13^ dexamethasone^14,15^ and triamcinolone acetonide^16^ respectively).

Only, the exact route to reach the posterior segment following topical administration is extremely complex, featuring plenty of static/dynamic barriers making it difficult to model the whole progress or to estimate the amount of drug reaching posterior tissues. Drug levels after topical administration in the vitreous and retina-choroid has been studied by in vivo and ex vivo methods. In vivo methods are expensive and unethical, as they include ‘simple’ pharmacokinetic studies requiring the execution of the animals for each time point or vitreous dialysis involving surgical placement of a probe into the rabbit’s eye and eventually, execution of the animal after the experiments^6,7,9,17–19^. In the survey of *Maurice et al.* these methods are criticized because tissue contamination can occur during enucleation due to eye drop residues on the lid margins and eyelashes, meanwhile drug redistribution after death may also be an issue^20^. Ex vivo methods usually include arterial perfusion of the mammalian eye^10,21^ or monitoring the distribution of a fluorescent compound by optical coherence tomography^16^. The main issue is that all in vivo and ex vivo studies are either purely methodological or focus solely on a single drug substance, resulting in limited data on the posterior segment permeability of a few drugs following topical administration. In vitro methods could be cost-effective, high-throughput and robust, but to the present, in vitro studies focus exclusively on intravitreal injections, investigating the diffusion of drugs in various polymer hydrogels mimicking the vitreous humor^22–25^.

This study aims to develop a permeability model utilising the PAMPA (Parallel Artificial Membrane Permeability Assay) method to evaluate posterior segment permeability followed by topical administration. Modification of the corneal-PAMPA model (focusing on the corneal permeability process) by altering the acceptor phase to mimic the vitreous body hopefully allows us to model the permeability process starting from the eye surface targeting the vitreous humor. Due to the involvement of many tissues including cornea, conjunctiva, sclera, iris, lens, vitreous, and choroid in the entire process, no precise permeation route is reported. The study includes the investigation of a vitreous humor-mimetic material as acceptor-side medium validated by *Thakur et al.* for in vitro drug and particle migration, and physicochemical similarities with porcine vitreous humor are demonstrated by rheological characterization and zeta potential measurement.

## Experimental

### Materials

The active pharmaceutical ingredients such as antipyrine, benzocaine, buspirone hydrochloride, carbamazepine, cetirizine dihydrochloride, chloroquine diphosphate, desipramine hydrochloride, dexamethasone, diclofenac Na, diltiazem hydrochloride, duloxetine hydrochloride, flurbiprofen, enoxacin, hydrocortisone, ketorolac, lornoxicam, methapyrilene, moxifloxacin hydrochloride, nepafenac, norfloxacin, ofloxacin, oxybuprocaine hydrochloride, piroxicam, prednisolone, propamidine, propranolol hydrochloride, pyrilamine, ranitidine hydrochloride, tenoxicam, tetracaine hydrochloride, timolol maleate and other chemicals like phosphate buffered saline (PBS) powder, L-α-phosphatidylcholine (PC) were purchased from Merck KGaA (Darmstadt, Germany). Analytical grade solvents such as dimethyl sulfoxide (DMSO), acetonitrile, methanol, chloroform, n-hexane, dodecane were purchased also from Merck KGaA. Hyaluronic acid sodium salt (Na-HA) with different molecular weights: 200 kDa (HA-200) and 855 kDa (HA-855) was accessible from Medicinal Chemistry Laboratory II, Gedeon Richter Plc, (Gyömrői Street 19–21, 1107 Budapest, Hungary) with Erika Forrai’s assistance. Agar powder was purchased from VWR International Ltd. (Radnor, Pennsylvania, USA). In all experiments, distilled water was purified by the Millipore Milli-Q^®^ 140 Gradient Water Purification System. To gain freshly excised vitreous humor, porcine eyes were obtained from a local slaughterhouse (Porció-ÉK Ltd., Albertirsa, Hungary).

### Preparation of hyaluronate and agar-based solutions

To prepare HA/agar solutions the exact amount of HA and/or agar powder was weighted, added into boiling PBS (pH 7.4) and completely dissolved by vigorous stirring for 15 min. Finally, the solutions were stored at room temperature overnight.

### In vitro permeability measurements (PAMPA)

For the parallel artificial membrane permeability assay (PAMPA) previously published method of corneal-PAMPA was modified in some parameters^26^. First, the active pharmaceutical ingredients (APIs) were dissolved in PBS buffer (pH 7.4) to make solutions of 250 µM nominal concentration, except for two drugs: carbamazepine and dexamethasone, which were used in 100 µM nominal concentration because of their poor solubility. Before each assay the PBS stock solutions were homogenized by using a Boeco Vortex Mixer V1 plus for 10–12 s and an ultrasonic bath (VWR Ultrasonic Cleaner) for 10 min. After that, phosphatidylcholine (PC, 16 mg) was dissolved in a solvent mixture (600 µL) of 70% (v/v) hexane, 25% (v/v) dodecane, 5% (v/v) chloroform at 0 °C using an ultrasonic bath. Then 5 µL of the lipid solution was pipetted into each well of donor plate (MultiscreenTM-IP, MAIPN4510, pore size 0.45 mm; Millipore), creating an artificial membrane of PC/dodecane solution on the porous bottom of the wells as hexane and chloroform evaporated. Then the donor plate was fit into the acceptor plate (Multiscreen Acceptor Plate, MSSACCEPTOR; Millipore), already containing 300 µL acceptor phase solution (PBS or polymer solution). Finally, 150 µL of the API solutions were put on the membrane of the donor plate. For each assay 3 replicates per compounds were measured. The sandwich plate was covered with a sheet of wet paper tissue and a plate lid to avoid evaporation of the solvent. The plates were incubated for 4 h at 35 °C (Heidolph Titramax 1000), followed by separation of the plates and analyzation of the acceptor phase by UV-vis plate reader (see at Sect. ***2.6 UV-vis spectroscopy***). Absorbance data of the ‘theoretical equilibrium’ was also collected using the so called equilibrium solutions prepared as a mixture of 100 µL acceptor phase solution and 50 µL API solution.

For calculating apparent permeability based on absorbance data, the following equation was used published by Faller et al.^27^:

1 $$\:{P}_{a}=-\frac{\text{V}\text{D}\times\:\text{V}\text{A}}{\left(\text{V}\text{D}+\text{V}\text{A}\:\right)\times\:\text{A}\times\:\text{t}}\times\:\text{ln}\left(1-r\right)$$

where *P*_a_ is the apparent permeability coefficient (cm/s), *V*_*D*_ and *V*_*A*_ are the volumes in the donor (0.15 cm^3^) and acceptor phase (0.3 cm^3^), *A* is the filter area (0.24 cm^2^), *t* is the incubation time (14400 s) and $\:\:r=\frac{\left[\text{A}\text{b}\text{s}\right]\text{A}\text{c}\text{c}\text{e}\text{p}\text{t}\text{o}\text{r}}{\left[\text{A}\text{b}\text{s}\right]\text{E}\text{q}\text{u}\text{l}\text{i}\text{b}\text{r}\text{i}\text{u}\text{m}}$, where *Abs* is the absorbance of acceptor and equilibrium solutions.

### Viscosity measurement

Complex viscosity of acceptor phase solutions was measured using an Anton Paar Physica MCR301 (Anton Paar GmbH, Graz, Austria) rheometer in oscillatory mode. Temperature was kept at 35 ± 0.1 °C by a Peltier device during all measurements. A parallel-plate plate geometry was used with a probe diameter of 25 mm (PP25). In each test, 100 µL of the formulations was introduced on the lower plate, and each formulation was measured in triplicate. Complex viscosity was recorded at a strain (γ) of 3% in the angular frequency (ω) range of 100 –0.1 rad/s with measuring three data points per decade.

### Zeta potential measurement

Zeta-potential of the acceptor phase solutions was measured using a Malvern Panalitycal ZetaSizer ProBlue instrument equipped with a 633 nm red laser at a detection angle of 173° (back-scattering mode) (Malvern Panalytical Ltd, Malvern, UK). Measurements were performed in 1 × 1 cm cuvettes (DTS0012) in triplicates applying a DIP cell (ZEN1002) probe at 35.0 °C. For data analysis ZS Explorer software was used.

### UV–Vis spectroscopy

Absorbance data were collected by a Multiskan Sky UV plate reader (ThermoFisher Scientific Inc., Waltham, USA; performance parameters are available in the ***Supplementary Material Table S5.***) 150 µL of acceptor and equilibrium solution were placed into the wells of a 96-well UV-Star plate (Greiner Bio-One Hungary Kft, Budapest, Hungary), then the plate was scanned on a wavelength range of 200–500 nm with 1 nm steps. For permeability calculations, absorbance data measured at the λ_max_ of each compound was used (limits of detection and quantification are available in the ***Supplementary Material Table S4.***).

### Statistical analysis

Graphpad Prism (v. 8.01) software was used to carry out statistical analysis and to create graphs. Two-way ANOVA test with Dunnett’s multiple comparisons test was used to determine significant differences at 95% confidence level.

## Results and discussion

### Physicochemical characterization of the compound set

**Fig. 1:**
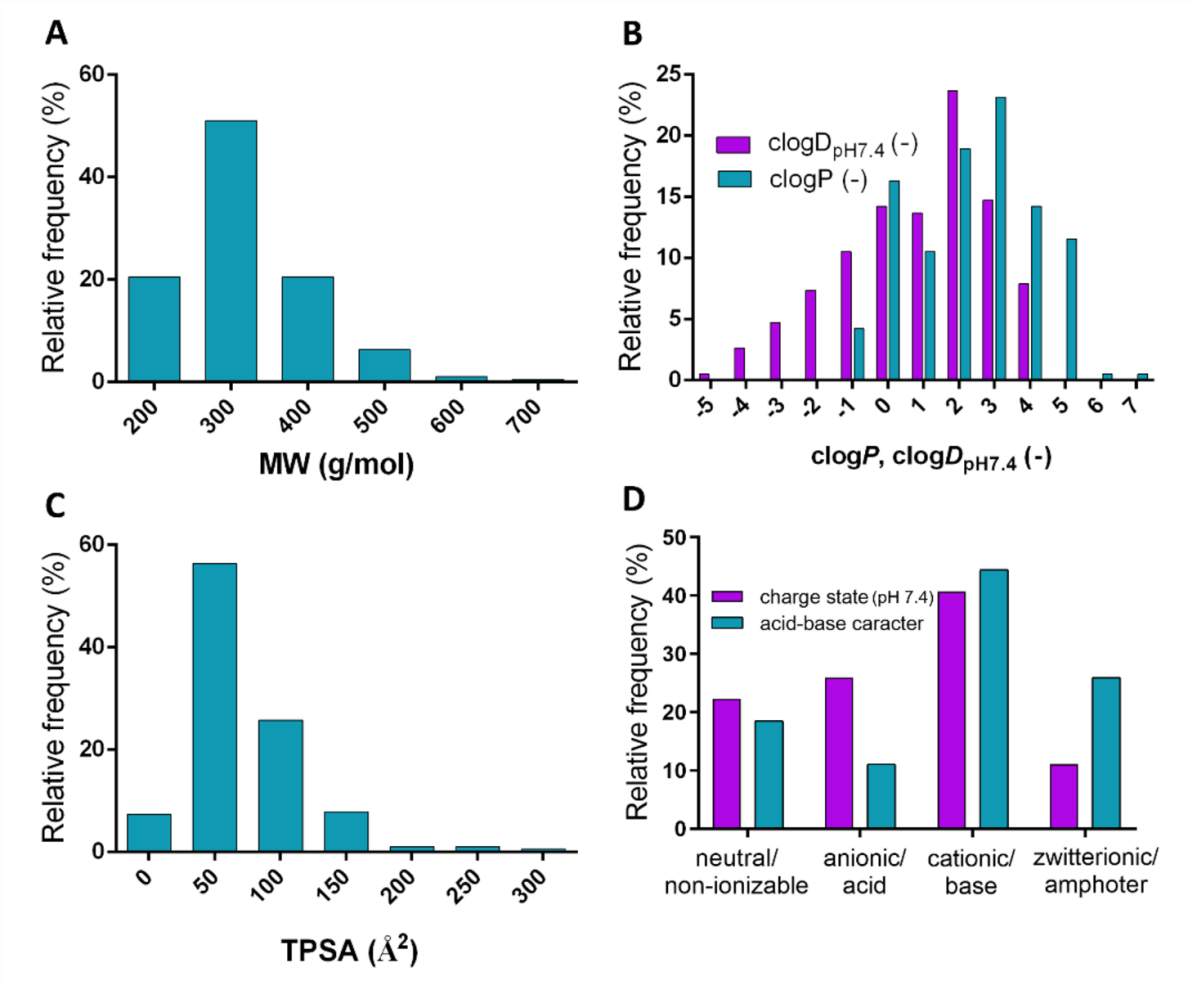
Frequency distribution of the investigated compounds’ physicochemical properties (*n* = 27). (**A**) Molecular weight, (**B**) lipophilicity, (**C**) topological polar surface area, (**D**) charge state at pH 7.4 and acid-base character (based on p*K*_a_ values listed in the Supplementary Material, Table S1.). All parameters were calculated using the ACD/Percepta software^28^.

For the model development, 27 compounds were selected with various physicochemical properties. Figure 1. shows the frequency distribution of molecular weight (MW), lipophilicity (log*P*, log*D*_pH7.4_), TPSA and charge state at pH 7.4 of the compounds.

The histograms show the classic range of properties for small molecules according to Lipinski’s and Veber’s rules: molecular weight less than 500 g/mol, log*P*/log*D*_pH7.4_ less than 5, TPSA less than 150 A^2^^29,30^. In this study, the compounds were classified based on their charge states at pH 7.4, considering the possible interaction with negatively charged polymer components (HA, agar) on the acceptor side. The compounds’ frequency distribution exhibits a well-known trend, with the majority of compounds being basic (mostly carrying a positive charge at pH 7.4).

### Corneal-PAMPA model translation with new analytical method

The corneal-PAMPA method published by our group in 2019 was introduced as a non-cell based model to predict corneal permeability of topically applied drugs^26^ and later it was used to build a permeability dataset for developing a new in silico predictive model for corneal permeability^31^. In the original setup, HPLC was used as analytical method, but unfortunately, the new setup designed for the posterior segment involves the use of HA which presents some challenges: the removal of HA from a few hundred microliter sample is difficult and wasteful, while not removing it could potentially cause damage to the instrument due to the pH-sensitive viscosity of HA^32^. To solve this problem, UV-vis plate reader (as a direct HTS analysis without sample preparation steps) was applied, and therefore a new calculation method was required to gain permeability data due to the lower sensitivity of this instrumentation. *Faller et al.* published a PAMPA method supported by an UV-vis plate reader, which involves a simpler equation to calculate apparent permeability (*P*_a,_ Eq. (1)). In this case only the absorbance data from the acceptor phase and the theoretical equilibrium (initial donor and acceptor solution mixed in 1:2 ratio) is to be collected.

**Fig. 2:**
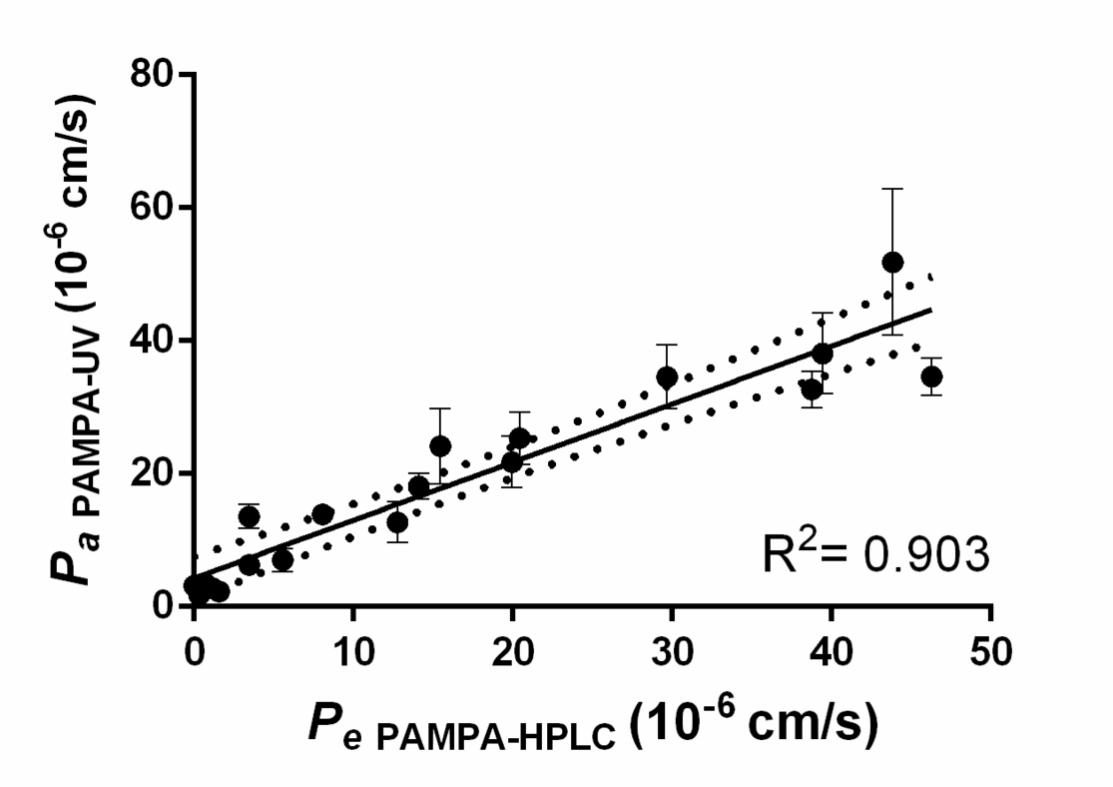
Corneal-PAMPA model translation (*n* = 19)—linear correlation between permeability values obtained from the original corneal-PAMPA (abscissa) and the newly introduced modified PAMPA model (ordinate).

To demonstrate the applicability of the new analytical and calculating method, 19 molecules were measured with the modified corneal-PAMPA method using higher drug solution concentrations (250 µM) due to the new method’s lower sensitivity (UV-vis plate reader). As shown in Fig. 2., newly obtained permeability values (*P*_a, PAMPA−UV_) exhibited a strong correlation with the values obtained from prior corneal-PAMPA measurements using HPLC (*P*_e, PAMPA−HPLC_, R^2^ = 0.903).

Based on this, the corneal-PAMPA method was successfully translated to the new analytical method required further on for detection of drugs in the HA-containing media.

### Development of the Vitreous-PAMPA model

**Fig. 3:**
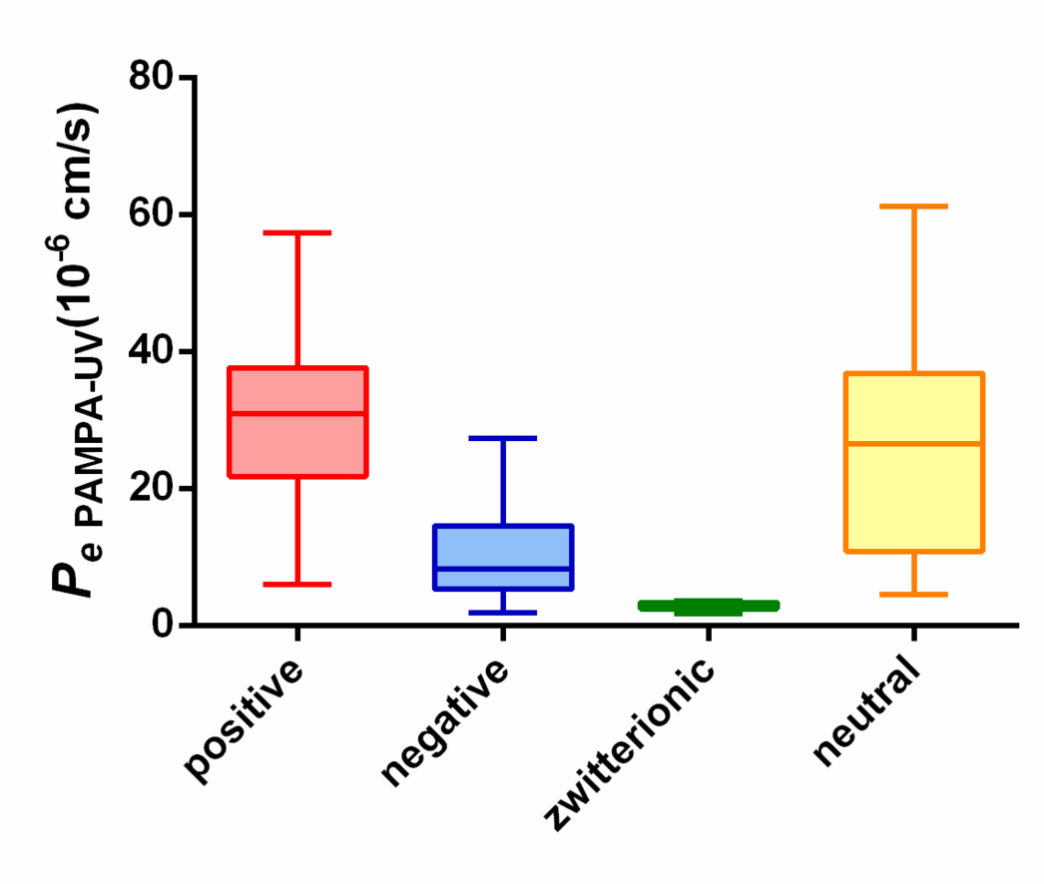
Box plot of compounds’ permeability in the models based on charge state (*n* = 27).

For developing a novel permeability model, the acceptor medium was systematically modified to mimic the VH. Meanwhile, general trends were observed regardless the acceptor medium, as neutral and positively charged APIs typically exhibited higher permeability compared to zwitterionic and negatively charged ones (Fig. 3.).

It is probable that the stable trends arise from the constant membrane composition (phosphatidylcholine in all cases), which is a crucial model parameter in PAMPA. Most cases eventually showed detectable (low to moderate) shifts in permeability. Henceforth, the focus is on the direction and magnitude of shifts rather than precise permeability values (details are available in the ***Supplementary Material***,*** in Figure ******S1******.***).

#### Permeability of compounds using HA on the acceptor side

**Fig. 4:**
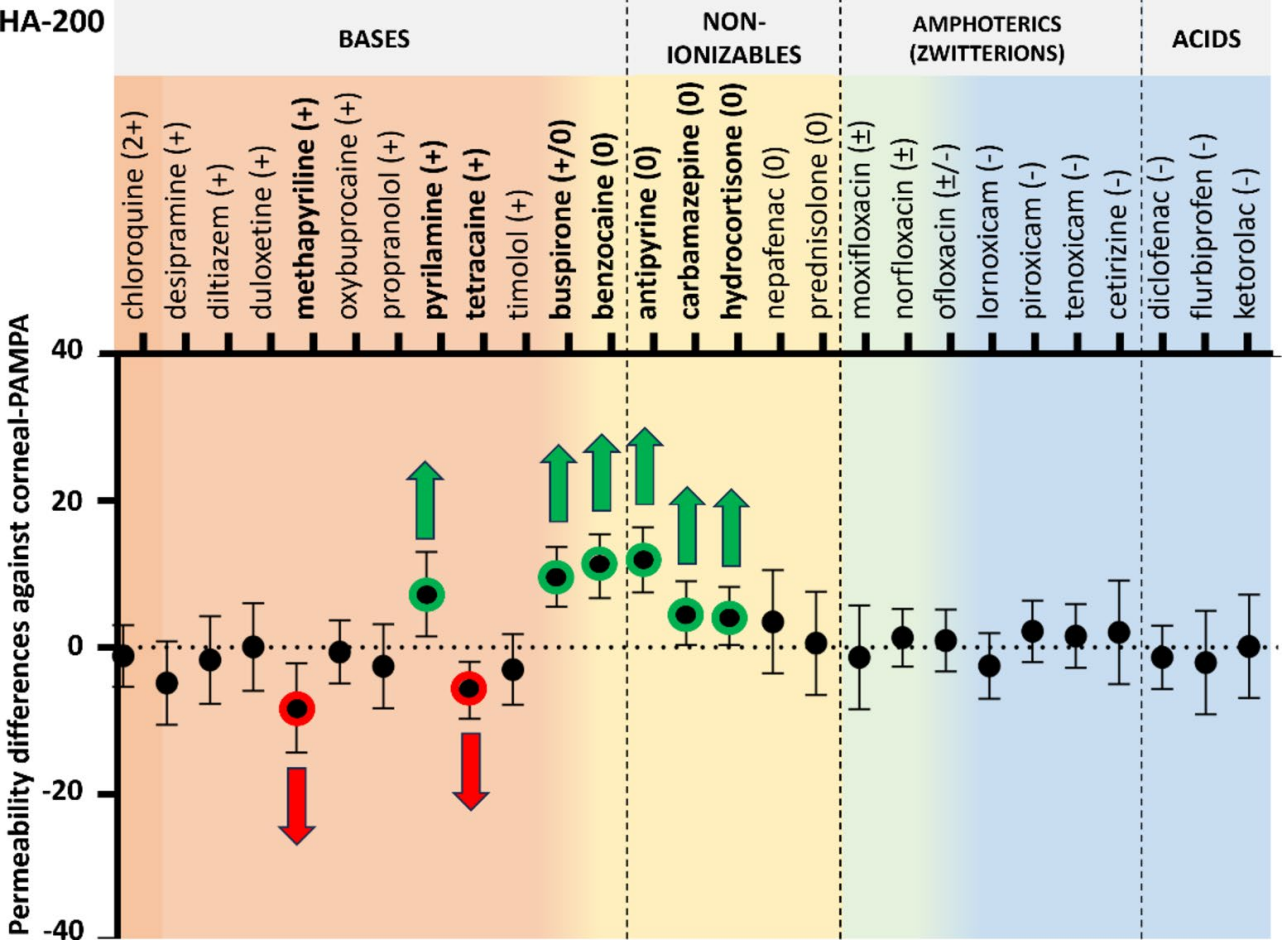
Permeability differences with 95% confidence intervals against corneal-PAMPA (PBS) vs. HA-200 (0.1 w/v%) as acceptor medium. Significant differences (two-way ANOVA, Dunnett’s multiple comparisons test, *p* < 0.05, *n* ≥ 3) are marked with red (decrease) and green (increase) arrows.

**Fig. 5:**
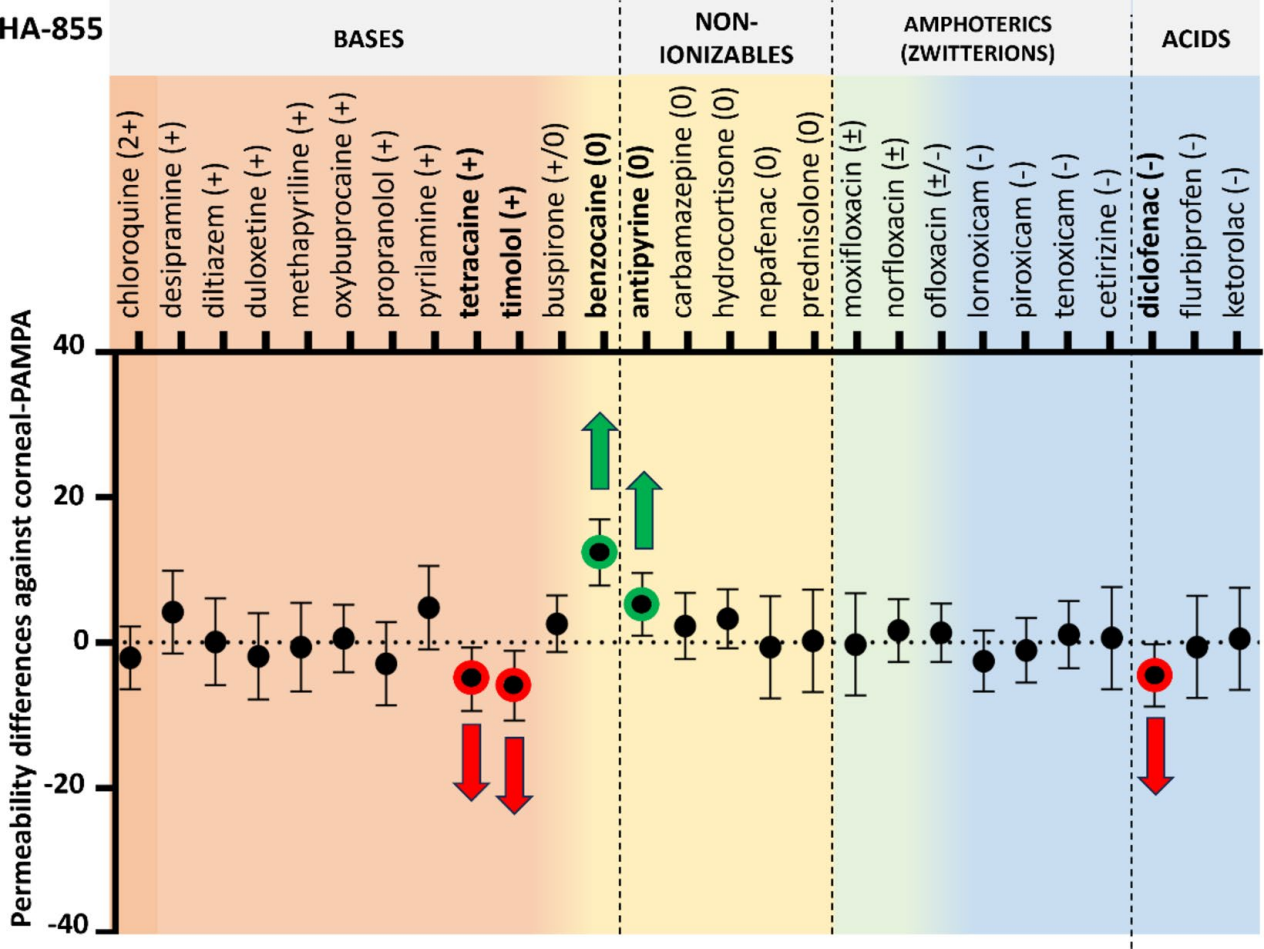
Permeability differences with 95% confidence intervals against corneal-PAMPA (PBS) vs. HA-855 (0.1 w/v%) as acceptor medium. Significant differences (two-way ANOVA, Dunnett’s multiple comparisons test, *p* < 0.05, *n* ≥ 3) are marked with red (decrease) and green(increase) arrows.

In the first step, HA was added to the corneal-PAMPA model as acceptor-phase component. Two types of Na-HA were used in the experiments at 0.1 w/v% concentration: mid molecular weight (200 kDa, HA-200) and high molecular weight HA (855 kDa, HA-855). Compared to the corneal-PAMPA model translation, 4 APIs had to be removed from the experiment as they could not be detected in the acceptor medium (due to very low permeability and the interference of HA with their absorbance signal: dexamethasone, enoxacin, propamidine, ranitidine). The remaining set was expanded with an additional 12 compounds and compared to the permeability values measured with the corneal-PAMPA model (*n* = 27). According to two-way ANOVA followed by Dunnett’s post hoc test, the presence of HA (regardless of molecular weight) did not affect the permeability values significantly in most cases compared to the corneal-PAMPA (PBS as acceptor media) permeability (Fig. 4. and Fig. 5.; ANOVA results can be found in the ***Supplementary Material***,*** Table S2.***). In six unique cases for HA-200, and for two cases for HA-855 a significant increase, meanwhile in three and two cases a significant decrease in permeability was measured respectively. Permeability increases in these cases were mostly associated with neutral compounds, while slight decrease was observed for a few basic compounds.

It is known that drugs applied topically on the eye cannot reach the VH in the same extent as they reach the aqueous humor^3^. Considering this, a model predicting significantly lower permeability compared to the corneal-PAMPA (which models the penetration toward the aqueous humor) would be more realistic.

#### Permeability of compounds with HA and agar on the acceptor side

In 2020, *Thakur et al.* published a paper about HA and agar-based hydrogels as suitable VH mimetics for in vitro drug and particle migration evaluations^33^. In their study three types of HA-agar hydrogels were investigated with various HA and agar content from rheological and particle migration aspects. Hyaluronate as a viscoelastic polymer is an essential part of the model (as a native component of the VH itself) while agar is used to achieve elasticity and so to mimic the properties of the naturally occurring collagen fibrils^24^. As a result of their study, they found the hydrogel containing 0.7 mg/ml (0.07 w/v%) HA (high molecular weight) and 0.95 mg/ml (0.095 w/v%) agar to be the most similar to bovine VH in the above-mentioned experiments. Since they conducted no permeability measurements with this promising hydrogel, we tested it as an acceptor medium in the PAMPA measurements. To gain further insight, experiments using only agar-containing acceptor phase were also carried out, since the standalone effect of the high molecular weight Na-HA was already investigated.

**Fig. 6:**
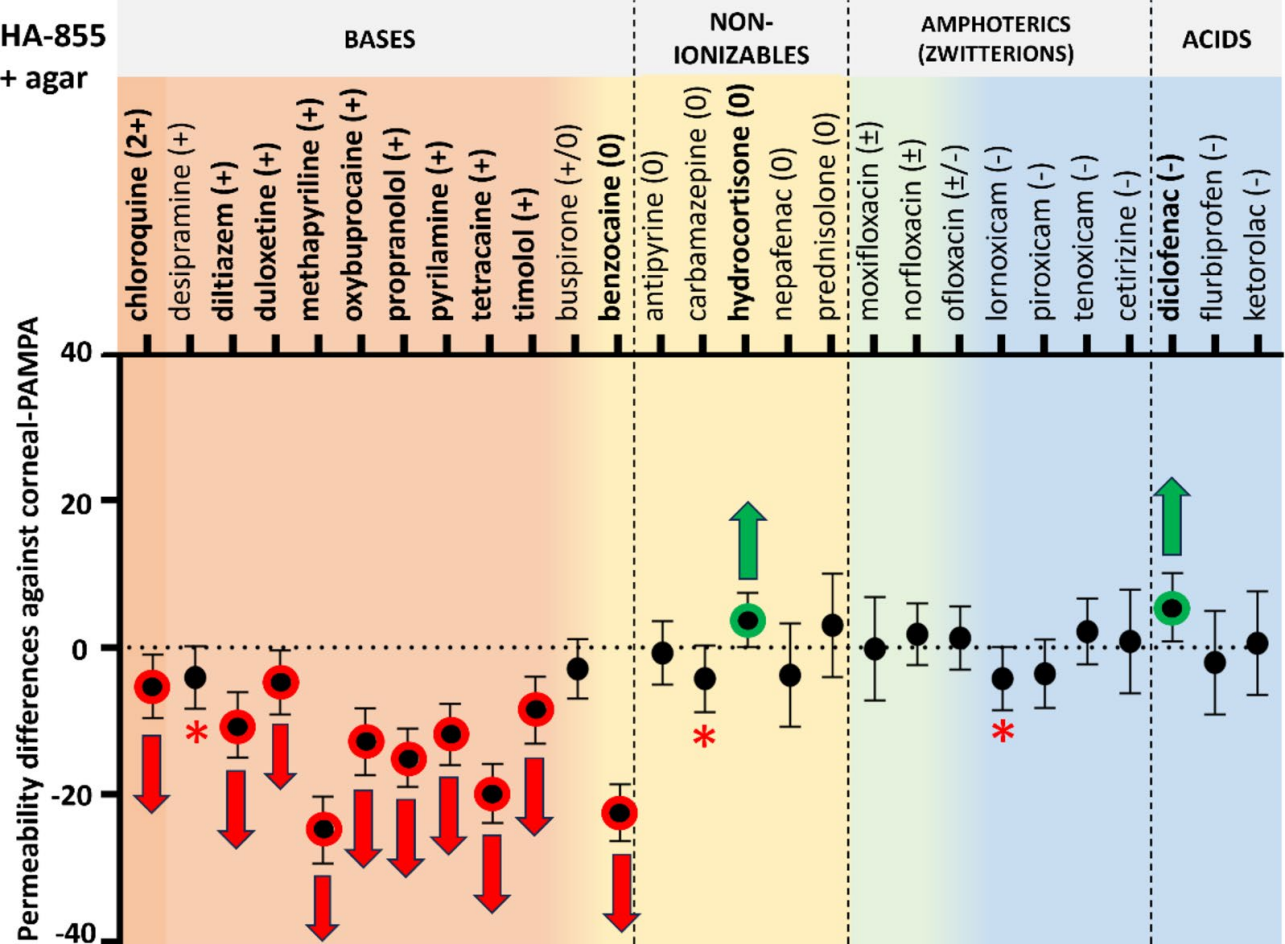
Permeability differences with 95% confidence intervals against corneal-PAMPA (PBS) vs. HA-855 + agar (HA:0.07 w/v%; agar: 0.095 w/v%) as acceptor medium. Significant differences (two-way ANOVA, Dunnett’s multiple comparisons test, *p* < 0.05, *n* ≥ 3) are marked with red (decrease) and green (increase) arrows. Compounds with p$\:\approx\:$0.05 are marked with red asterisks. Details on statistical analysis can be found in the Supplementary Material, Table S2.

**Fig. 7:**
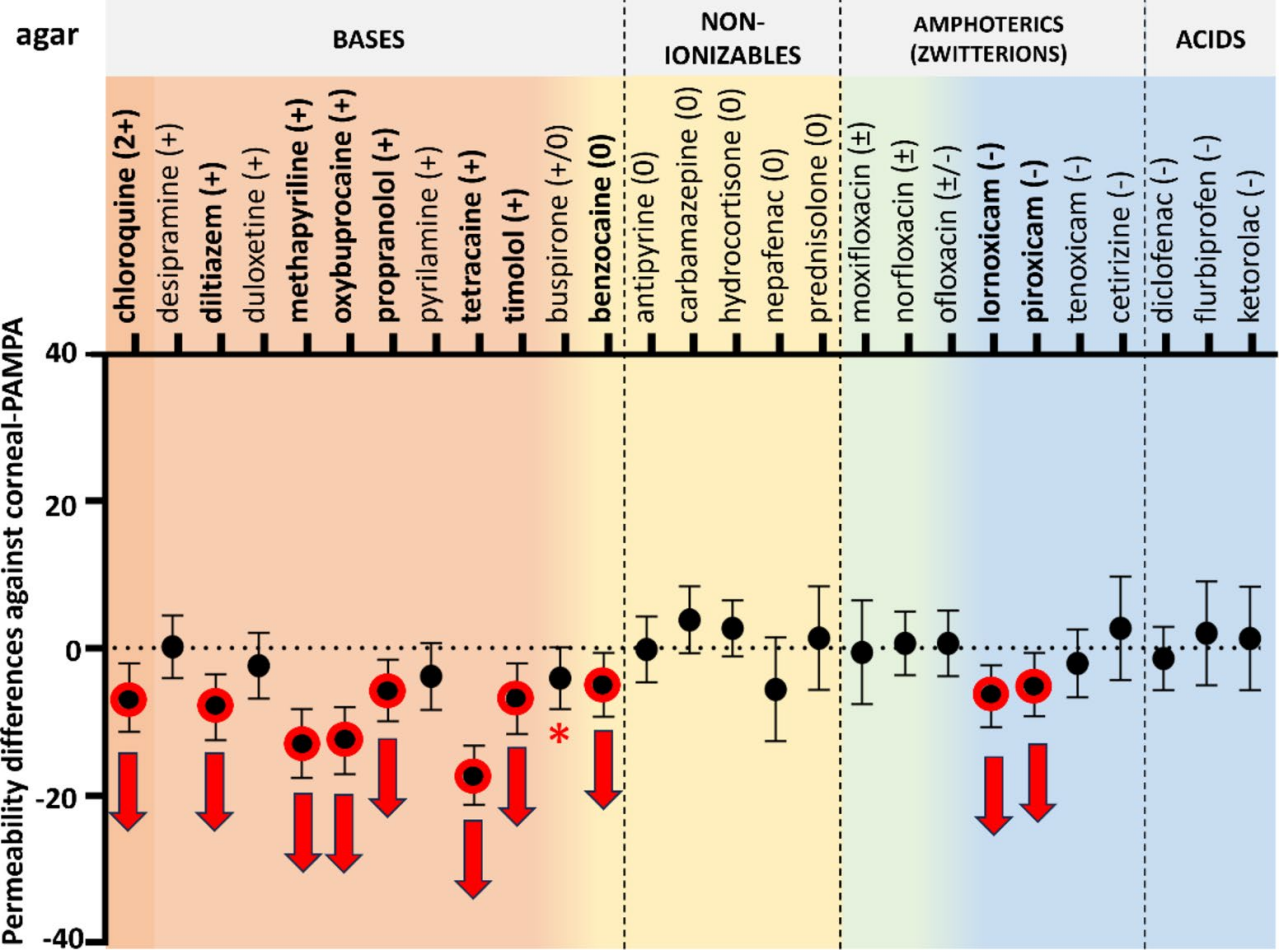
Permeability differences with 95% confidence intervals against corneal-PAMPA (PBS) vs. agar (0.095 w/v%) as acceptor medium. Significant differences (two-way ANOVA, Dunnett’s multiple comparisons test, *p* < 0.05, *n* ≥ 3) are marked with red (decrease) and green (increase) arrows. Compounds with p$\:\approx\:$0.05 are marked with red asterisks. Details on statistical analysis can be found in the Supplementary Material, Table S2.

As the results show, introduction of agar to the system significantly decreased the permeability of nearly all drugs with positive charge (~ bases) meanwhile, for other charge categories only small differences could be obtained (significant increase for hydrocortisone and diclofenac) (Fig. 6.). On the other hand, in the case of agar model, significant increase had not been observed for any compounds, moreover, two anionic compounds showed significant decrease in permeability (Fig. 7.). In general, the permeability values of negative and zwitterionic compounds are lower, therefore smaller differences can be recognized as significant ones. Nevertheless, examination of the figures reveals that the position of the negatively charged and zwitterionic datapoints were not changed that much (perhaps approaching the threshold of statistical traceability). In summary for cationic compounds, HA-855 + agar could decrease the permeability in a greater extent than agar alone (chloroquine, diltiazem, duloxetine, methapyrilene, oxybuprocaine, propranolol, pyrilamine, tetracaine, timolol).

According to a simple one-way ANOVA analysis followed by Dunnett’s post-hoc test, only HA-855 + agar and agar models showed significant differences (**, *p* < 0.01) in permeability, compared to PBS (See ***Supplementary Material***,*** Table S3.***)

### PAMPA with Porcine vitreous humor

**Fig. 8:**
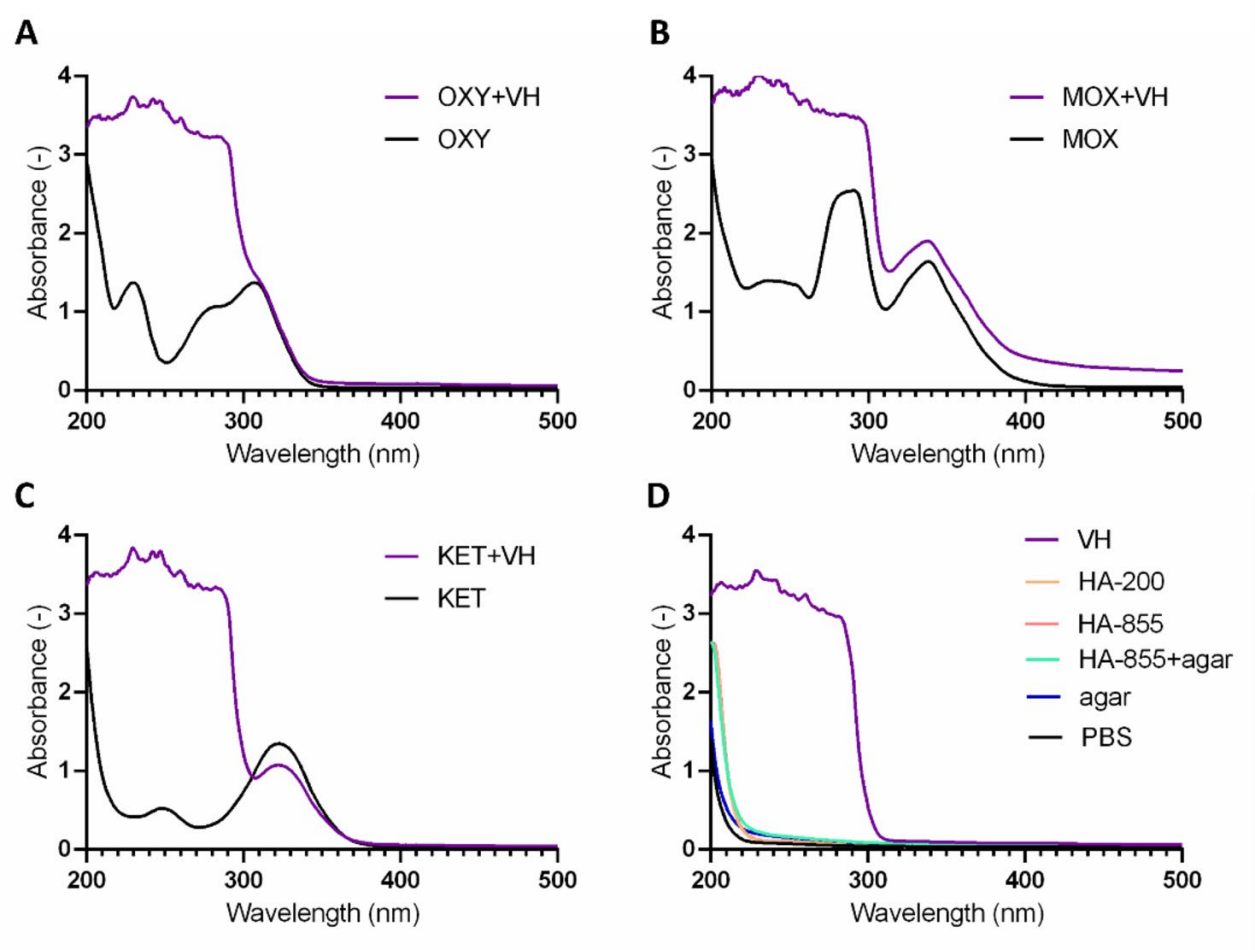
UV spectra of the equilibrium solutions (containing VH) vs. the initial API solutions (oxybuprocaine—**A**, moxifloxacin—**B**, ketorolac—**C**), and spectra of the various acceptor media (**D**). After removing protein components by precipitation and filtering, no API could be detected.

There has been an attempt made to use freshly excised porcine vitreous as acceptor medium in the experiments, for three APIs with sufficient absorption at a wavelength closer to the visible range (~ 300 nm) considering the cut-off of VH. Beyond the issue of inhomogeneous VH samples (hence the inaccuracies in pipetting) no significant signal (or non-noise signal indicating the presence of an API) could be found in the acceptor wells, which could be the result of the high UV absorption of the VH itself. When preparing the equilibrium solutions, one unit of API solution (250 µM, PBS) was mixed with (approximately) two units of VH. Figure 8**/A-C.** shows the spectra of the mixed samples along with the individual spectra of the APIs. Two APIs (ketorolac, moxifloxacin) absorption peaks could be observed, at 341 and 320 nm respectively, however these components are characterized with low permeability due to their acidic/amphoteric properties, therefore their possible absence on the acceptor side is reasonable. To gain clear spectra by removing background components, precipitation was attempted using cold acetonitrile, centrifugation, and filtration. However, no APIs could be detected in the filtered material, possibly due to the detection limit or their loss along with some protein components during the process. The sample preparation process of VH containing samples was not further improved or investigated in this study.

As Fig. 8/D. shows, the background absorbance of the HA-agar medium is negligible compared to the VH, therefore it is a more suitable medium for rapid, spectroscopic detection of APIs.

### Physicochemical characterization of the acceptor media

To further characterize the acceptor media and to reveal possible phenomena possibly responsible for the changes in permeability, the solutions’ complex viscosity and zeta potential were measured and compared with those of freshly excised VH samples of porcine origin.

**Fig. 9:**
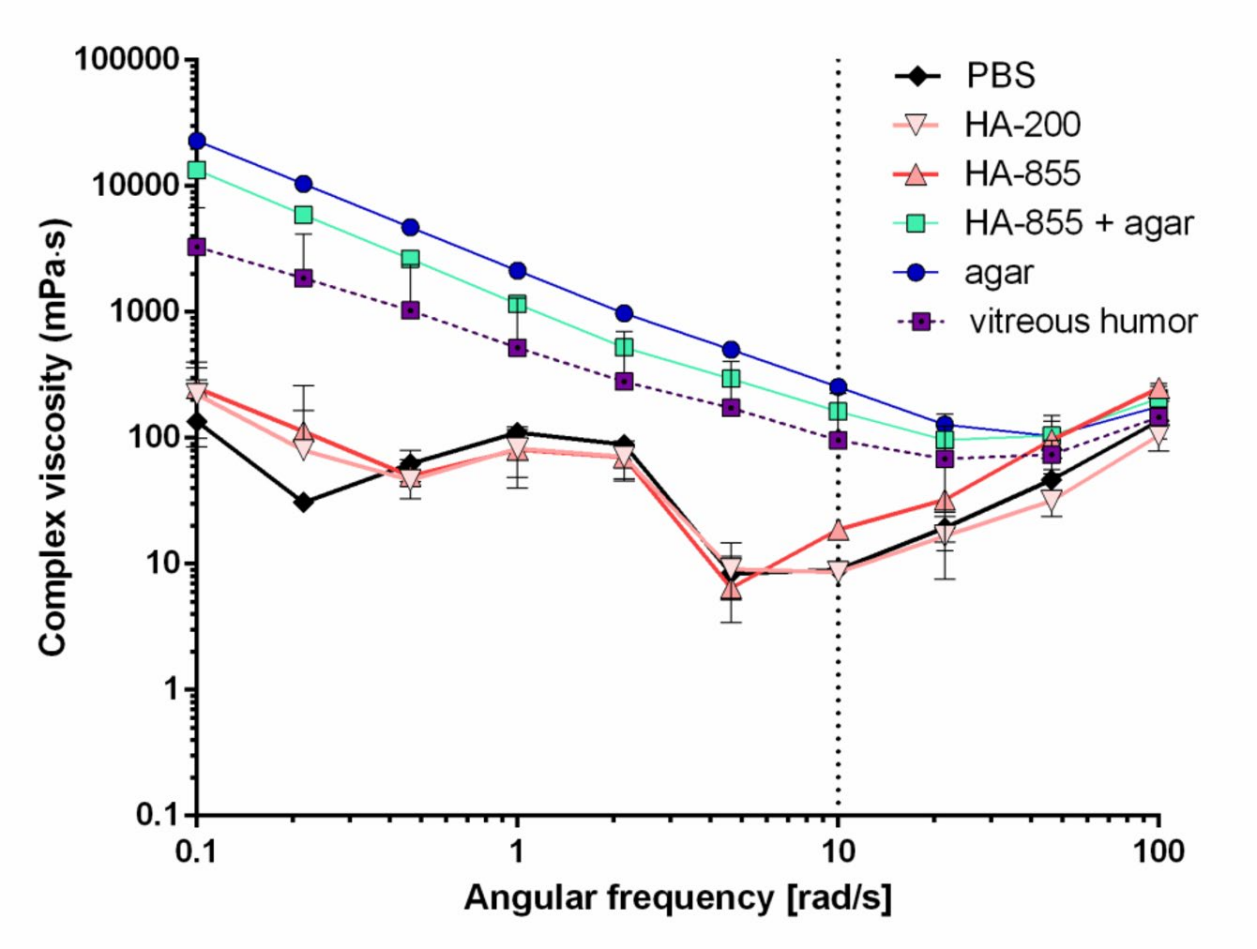
Complex viscosity of solutions used as acceptor media against angular frequency at 35 °C.

Acceptor media was characterized using a rheometer in oscillatory mode. Complex viscosity of the samples was plotted as the function of angular frequency in Fig. 9. On the plot, two groups came apart: PBS and HA solutions showed low complex viscosity meanwhile HA-855 + agar and agar-containing samples were found to be similar to the porcine VH, showing higher complex viscosities at low shear rates and a gradual decrease with the increasing shear rate.

**Fig. 10:**
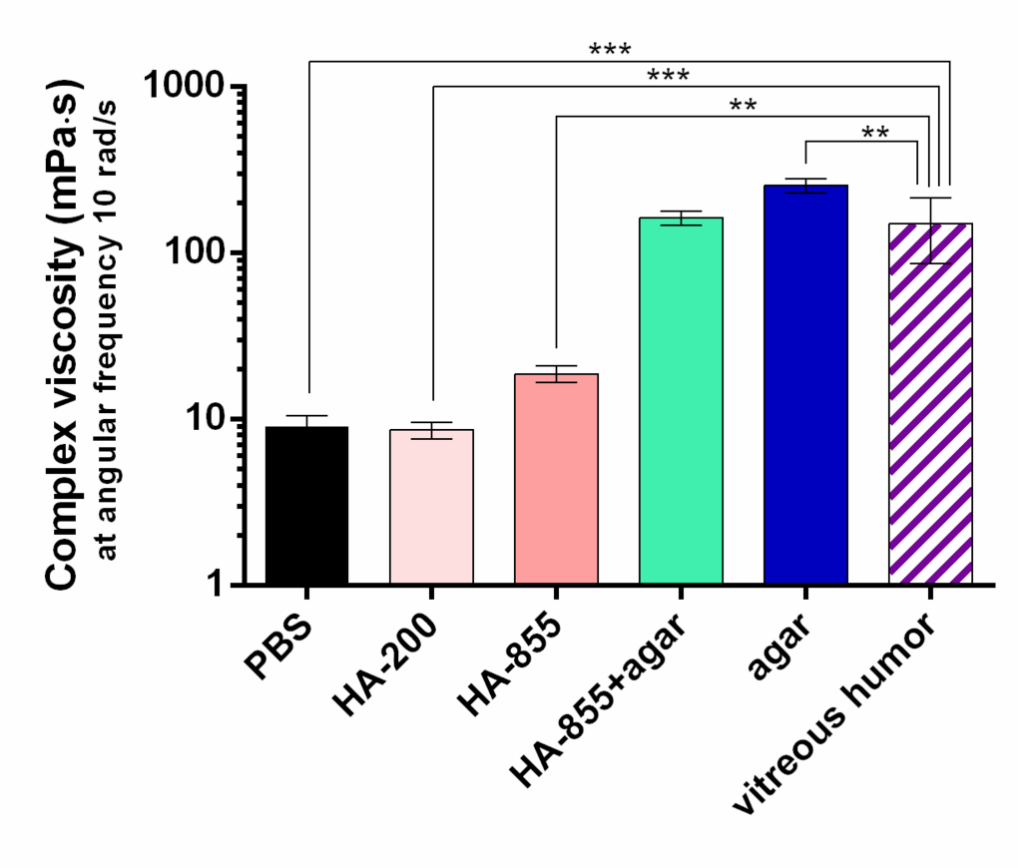
Complex viscosity of the solutions used as acceptor media at an angular frequency of 10 rad/s at 35 °C. Significant differences compared to the vitreous humor were revealed using one-way ANOVA with Dunnett’s multiple comparisons test and are marked with asterisks (**: *p* < 0.01, ***: *p* < 0.001).

Complex viscosity values obtained at 10 rad/s were further compared in Fig. 10., where except from HA-855 + agar, all samples showed significantly different complex viscosity compared to the VH. The values obtained for HA-855 + agar sample were similar to that reported by *Thakur et al.*., additionally, the porcine VH also displayed comparable viscosity to their bovine sample.

#### Zeta potential measurement

**Fig. 11:**
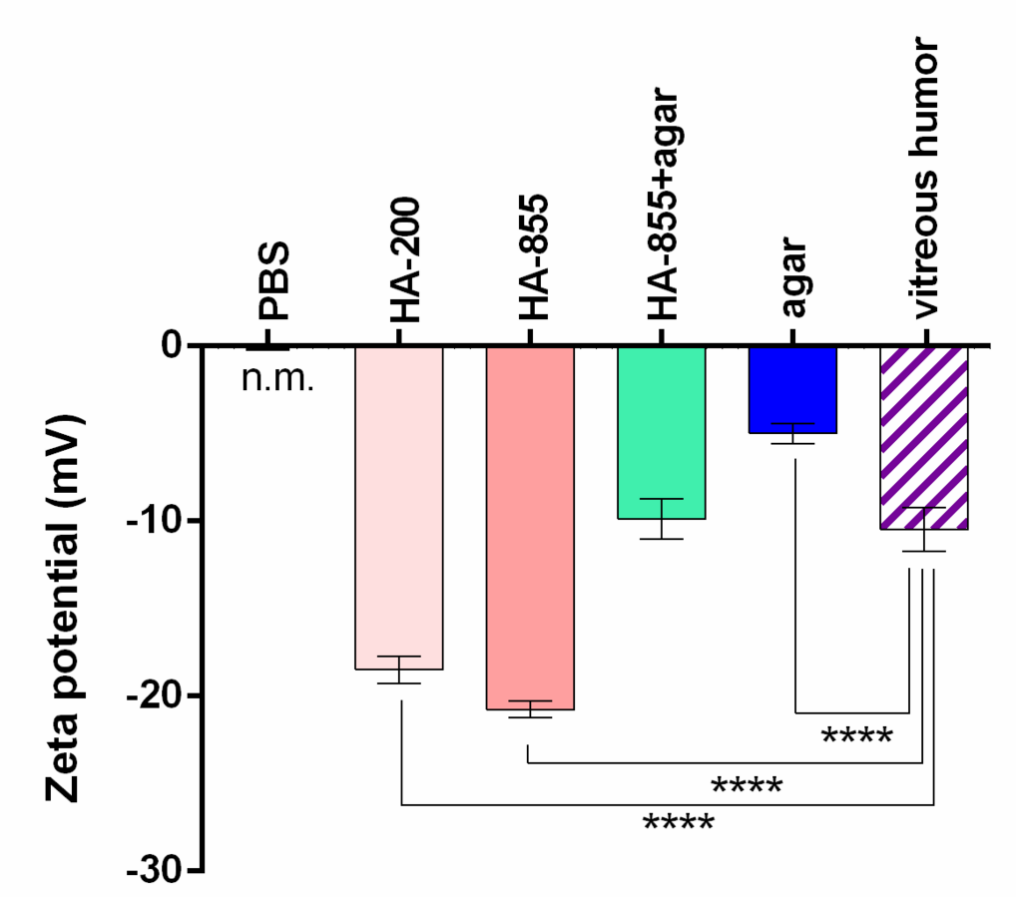
Zeta potential of various solutions used as acceptor media at 35 °C. Significant differences compared to the vitreous humor were revealed using one-way ANOVA with Dunnett’s multiple comparisons test and are marked with asterisks (****: *p* < 0.0001); n.m: not measurable.

Measuring zeta potential of polymer solutions can be helpful in the understanding of molecular interactions in aqueous medium. Zeta potentials of HA-containing solutions were found to be around − 20 mV, while HA-855 + agar and the VH itself showed much lower zeta potential (~ -10 mV). In fact, agar showed the second lowest zeta potential (~ -5 mV) as PBS hardly differed from zero, since particles are not present in this medium (Fig. 11.). According to the results, HA-855 + agar closely resembled porcine VH, possibly indicating similar electrolyte content and molecular interactions in solutions. Hyaluronates and agar are known to carry negative charges in solutions, moreover, the vitreous humor itself have been described as a negatively charged meshwork^34–36^. Studies on the migration of charged nanoparticles through the vitreous body revealed that anionic particles may freely diffuse through it, meanwhile cationic particles are trapped inside, regardless of their size [113, 117]. The fact, that in our case polymeric samples without agar showed higher negative zeta potentials suggests that other interactions may exist that influence the permeability of compounds in agar-containing models, apart from electrostatic ones.

In summary, a model based on corneal-PAMPA with modified acceptor medium (HA and agar containing solution) offers a promising approach for predicting the permeability of drugs toward the vitreous after topical administration. This is supported by findings indicating similarity in zeta potential and rheological behaviour to porcine vitreous humor, as well as consistent effects observed for charged molecules, aligning with previous reports. However, it’s essential to note that establishing a well-defined model would require proper validation, which is currently hindered by the limited availability of data on the vitreous-directed permeability of APIs.

## Conclusion

As a prelude of this work, *Thakur et al.* validated a Na-HA and agar containing hydrogel as suitable substitute of vitreous humor, possessing similar rheological properties, and allowing similar particle migration in this medium. In this study, the same hydrogel was tested in the in vitro PAMPA model as acceptor medium, and as a result, significantly lower permeability could be obtained compared to the corneal-PAMPA model, or the modified, HA-containing models. Further investigations revealed that this hydrogel can in fact be a good analogue to the vitreous body, as zeta potentials of this media and porcine vitreous body were found quite the same, which shows similar behaviour of their molecules in the solution phase.

The current model for posterior segment permeability may not be optimal due to the limited data available on the actual permeability of topically applied drugs toward the posterior segment. This lack of data hinders the ability to assess the similarity or validate the modell adequately. However, without improved predictive and high-throughput models, and considering the challenges of directly obtaining information from animal vitreous studies, this model could serve as the initial step toward predicting the posterior segment permeability of drugs applied topically on the eye without the involvement of laboratory animals.

## Electronic supplementary material

Below is the link to the electronic supplementary material.

Supplementary Material 1

## Data Availability

All data is available in the Supplementary Material.

## Abbreviations

APIs: Active pharmaceutical ingredients
HA: Hyaluronate
HA-200: Hyaluronate with molecular weight of 200 kDa
HA-855: Hyaluronate with molecular weight of 855 kDa
HPLC: Hight performance liquid chromatography
MW: Molecular weight
Na-HA: Sodium hyaluronate
PAMPA: Parallel artificial membrane permeability assay
PBS: Phosphate buffered saline
PC: Phosphatidylcholine
TPSA: Topological polar surface area
VH: Vitreous humor
