# Supplementary material for: Toward a high-throughput in vitro model for estimating vitreous humor permeability of topically applied drugs
Source: Sci Rep. 2025 Mar 13;15:8768. doi: 10.1038/s41598-025-93425-3 (PMC11906762; doi:10.1038/s41598-025-93425-3)
Supplement: Supplementary file 1 — Supplementary Material 1 [file 41598_2025_93425_MOESM1_ESM.pdf]

Supplementary Material for the manuscript titled

Toward a high-throughput in vitro model for estimating vitreous humor permeability of topically applied drugs

Anna Vincze[a,b*], Eszter Simon[c], Gábor Koplányi[d], József Gergely Stankovits[e], Diána Balogh-Weiser[d], Benjámin Gyarmati[e], Zoltán Zsolt Nagy[f], György T. Balogh[a,b,*]

[a] *Department of Pharmaceutical Chemistry, Semmelweis University, Hőgyes Endre Street 9., H-1092 Budapest, Hungary*

[b] *Center for Pharmacology and Drug Research & Development, Semmelweis University, Üllői Street 26. H-1092 Budapest, Hungary*

[c] *Department of Electronics Technology, Faculty of Electrical Engineering and Informatics, Budapest University of Technology and Economics, Műegyetem Quay 3., H-1111 Budapest, Hungary*

[d] *Department of Organic Chemistry and Technology, Faculty of Chemical Technology and Biotechnology, Budapest University of Technology and Economics, Műegyetem Quay 3., H-1111 Budapest, Hungary*

[e] *Department of Physical Chemistry and Materials Science, Faculty of Chemical Technology and Biotechnology, Budapest University of Technology and Economics, Műegyetem Quay 3., H-1111 Budapest, Hungary*

[f] *Department of Ophthalmology, Semmelweis University, Mária Street 39., H-1085 Budapest, Hungary*

**Corresponding authors: vincze.anna@semmelweis.hu (A. Vincze), balogh.gyorgy.tibor@semmelweis.hu (Gy. T. Balogh)*

Contents

Table S1. Calculated physicochemical properties of APIs investigated

Figure S1. PAMPA permeability of charged APIs applying different solutions as acceptor medium

Table S2. Two-way ANOVA followed by Dunnett's multiple comparisons test**:** significant differences in permeability by compounds and models against corneal-PAMPA (PBS)

Table S3. One-way ANOVA followed by Dunnett's multiple comparisons test: significant differences in permeability by models against corneal-PAMPA (PBS)

Table S4. Limit of detection (LOD) and limit of quantification (LOQ) of compounds in UV spectrophotometry

Table S5. Performance parameters of the UV microplate reader

Table S1. Calculated physicochemical properties of APIs investigated

Compound name	Molecular weight (g/mol)	TPSA (Å^2)*	clog$P$ (-)*	clog$D_{pH7.4}$ (-)*	cp$K_{a, basic}$ (-)*	cp$K_{a, acidic}$ (-)*	acid-base characteristic	net charge at pH 7.4*
antipyrine	188.23	23.55	0.72	0.72	-	-	non-ionizable	0
benzocaine	165.192	52.32	1.83	1.83	2.51	-	non-ionizable	0
buspirone HCl	385.512	69.64	3.04	2.68	7.55; 4.25	-	base	(+)/0
carbamazepine	236.274	46.33	2.28	2.28	-	-	non-ionizable	0
cetirizine 2 HCl	388.89	53.01	2.51	-0.56	6.71;2.10	3.27	acid	(-)
chloroquine diphosphate	319.88	28.16	4.56	1.57	10.47;8.22	-	base	(2+)
desipramine HCl	266.388	15.27	4.26	1.56	10.40	-	base	(+)
diclofenac Na	296.15	49.33	4.48	1.37	-	4.18	acid	(-)
diltiazem HCl	414.52	84.38	3.43	2.06	8.13	-	base	(+)
duloxetine HCl	297.42	49.5	4.03	1.53	10.02	-	base	(+)
flurbiprofen	244.24	37.3	3.82	0.68	-	4.14	acid	(-)
hydrocortisone	362.466	94.83	1.66	1.66	-	-	non-ionizable	0
ketorolac	255.27	59.3	2.58	-0.44	-	3.52	acid	(-)
lornoxicam	371.81	136.22	2.33	-1.17	4.55	1.37	acid	(-)
methapyrilene HCl	261.39	47.61	2.86	0.99	8.90;3.73	-	base	(+)
moxifloxacin	401.43	82.11	0.78	-1.72	9.91;2.03	6.12	amphoteric	(±)
nepafenac	254.28	86.16	1.38	1.38	-	-	non-ionizable	0
norfloxacin	319.336	72.88	-0.56	-3.04	8.69;2.98	6.25	amphoteric	(±)
ofloxacin	361.373	73.32	0.17	-2.08	7.38	5.98	amphoteric	(±)/(-)
oxybuprocaine HCl	308.42	64.79	3.74	1.97	9.16;2.37	-	base	(+)
piroxicam	331.35	107.98	2.22	-1.16	4.81	3.03	acid	(-)
prednisolone	360.44	94.83	1.66	1.66	-	-	non-ionizable	0
propranolol HCl	259.349	41.49	3.48	1.41	9.07	-	base	(+)
pyrilamine	285.391	28.6	3.04	1.08	9.02;4.02	-	base	(+)
tenoxicam	337.37	136.22	1.74	-1.75	4.67	1.55	acid	(-)
tetracaine HCl	264.369	41.57	3.3	2.26	8.24	-	base	(+)
timolol	316.42	107.98	1.53	-0.32	9.53	-	base	(+)

*calculated with ACD/Percepta software

Figure S1. PAMPA permeability of charged APIs applying different solutions as acceptor medium

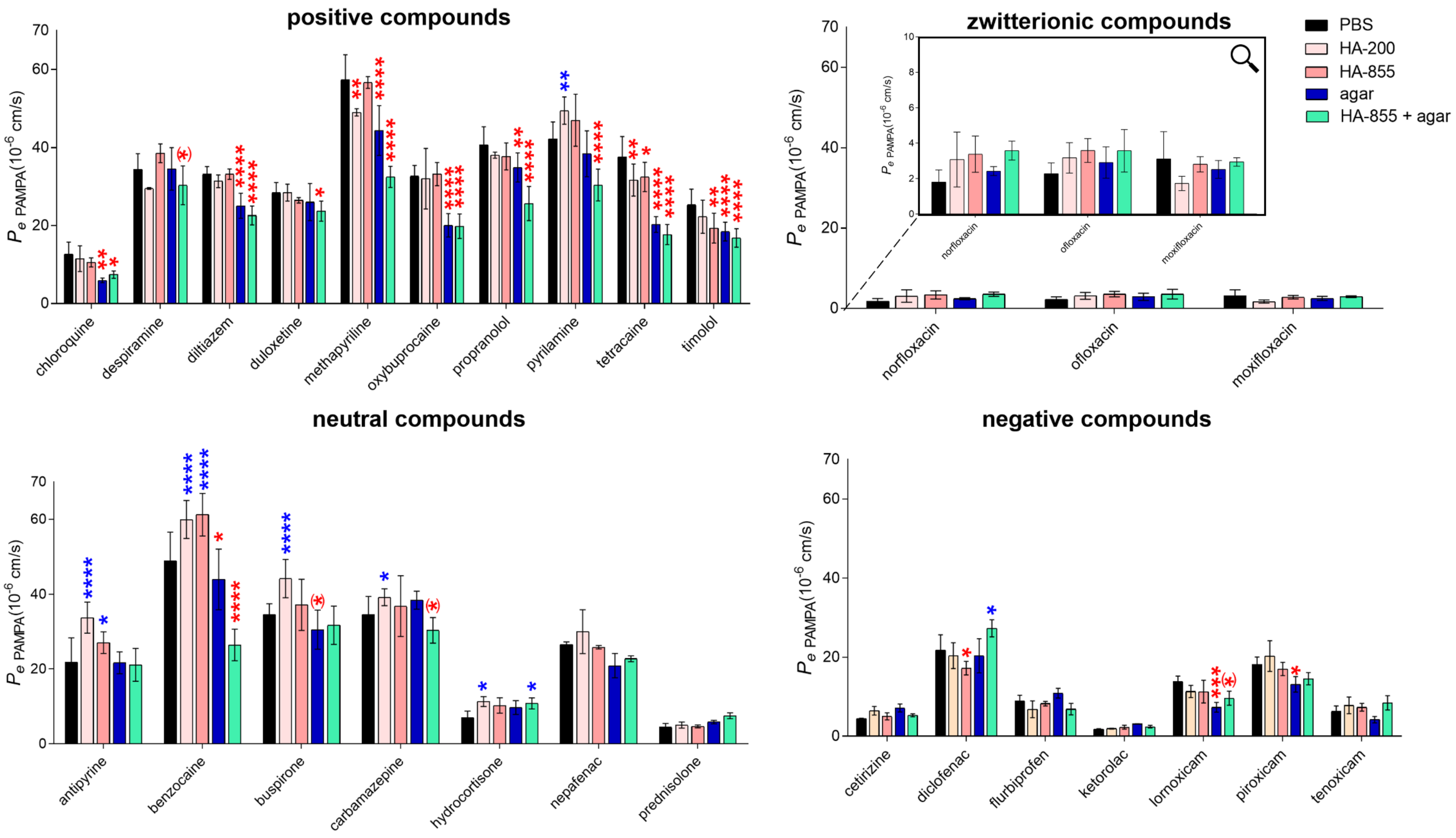

Table S2. Two-way ANOVA followed by Dunnett's multiple comparisons test**:** significant differences in permeability by compounds and models against corneal-PAMPA (PBS)

ns: not significant (with p value in brackets); * p<0.05; ** 0.05<p<0.01; *** 0.01<p<0.001; **** 0.001<p<0.0001

Compound name	HA-200	HA-855	HA-855+agar	agar
antipyrine	****	*	ns	ns
benzocaine	****	****	****	*
buspirone HCl	****	ns	ns	ns (0.057)
carbamazepine	*	ns	ns (0.073)	ns
cetirizine 2 HCl	ns	ns	ns	ns
chloroquine diphosphate	ns	ns	*	**
desipramine HCl	ns	ns	ns (0.062)	ns
diclofenac Na	ns	*	*	ns
diltiazem HCl	ns	ns	****	****
duloxetine HCl	ns	ns	*	ns
flurbiprofen	ns	ns	ns	ns
hydrocortisone	*	ns	*	ns
ketorolac	ns	ns	ns	ns
lornoxicam	ns	ns	ns (0.054)	***
methapyrilene HCl	**	ns	****	****
moxifloxacin	ns	ns	ns	ns
nepafenac	ns	ns	ns	ns
norfloxacin	ns	ns	ns	ns
ofloxacin	ns	ns	ns	ns
oxybuprocaine HCl	ns	ns	****	****
piroxicam	ns	ns	ns	*
prednisolone	ns	ns	ns	ns
propranolol HCl	ns	ns	****	**
pyrilamine	**	ns	****	ns
tenoxicam	ns	ns	ns	ns
tetracaine HCl	***	*	****	****
timolol	ns	**	****	****

Table S3. One-way ANOVA followed by Dunnett's multiple comparisons test: significant differences in permeability by models against corneal-PAMPA (PBS)

ns: not significant; ** $0.05<p<0.01$;

Comparisons	Mean Difference	95% CI of diff.	Significant?	Summary
PBS vs. HA-200	-0.9093	-3.338 to 1.519	No	ns
PBS vs. HA-855	-0.4147	-2.255 to 1.425	No	ns
PBS vs. HA-855 + agar	5.301	1.272 to 9.330	Yes	**
PBS vs. agar	3.412	0.7976 to 6.027	Yes	**

Table S4. Limit of detection (LOD) and limit of quantification (LOQ) of compounds in UV spectrophotometry

Compound name	LOD [μM]*	LOQ [μM]*
antipyrine	0.31	0.94
benzocaine	0.33	0.99
buspirone HCl	0.43	1.30
carbamazepine	0.40	1.21
cetirizine 2 HCl	1.91	5.76
chloroquine diphosphate	2.79	8.47
desipramine HCl	0.21	0.65
diclofenac Na	0.26	0.77
diltiazem HCl	1.43	6.18
duloxetine HCl	3.32	10.06
flurbiprofen	2.16	6.54
hydrocortisone	0.44	1.33
lornoxicam	3.16	9.56
ketorolac	2.07	6.29
methapyrilene HCl	2.02	6.10
moxifloxacin	2.67	8.06
nepafenac	5.23	15.83
norfloxacin	0.51	1.54

ofloxacin	2.84	8.62
oxybuprocaine HCl	0.59	1.79
piroxicam	3.77	11.41
prednisolone	3.26	9.83
propranolol HCl	1.51	4.56
pyrilamine	3.28	9.90
tenoxicam	0.29	0.88
tetracaine HCl	0.46	1.39
timolol	3.12	9.44

*LOD=3.3×σ /slope; LOQ=10×σ /slope; σ : the standard deviation of blank measurements, slope: the slope of the calibration line; absorbance data collected at the wavelength maximum for each compound

Table S5. Performance parameters of the UV microplate reader[1]

Instrument type	Multiskan Sky UV microplate reader (Thermofisher Scientific Inc.; Waltham, USA).
Linearity @ 450 nm	0–2.5 Abs, 2%
Accuracy @ 450 nm	1.0% + 0.003 Abs (0–2.0 Abs) 2.0% (2.0–2.5 Abs)
Precision @ 450 nm	SD < 0.003 Abs or CV < 1.0%

[1]Data available at the manufacturer's website. The specific values are valid for flat-bottom 96-well microplates
